# Reduction in risk of contrast-induced nephropathy in patients with chronic kidney disease and diabetes mellitus by enhanced external counterpulsation

**DOI:** 10.3389/fendo.2022.973452

**Published:** 2022-10-17

**Authors:** Chun-Mei Zeng, Yan-Mei Zhao, Xin-Jing Zhong, Zi-Jia Wu, Jing Bai, Shi-Yu Qiu, Yi-Yi Li

**Affiliations:** Department of Cardiology, Yulin First People’s Hospital (The Sixth Affiliated Hospital of Guangxi Medical University), Yulin, China

**Keywords:** enhanced external counterpulsation, chronic renal disease, diabetes mellitus, CAG/PCI, contrast-induced nephropathy

## Abstract

**Objective:**

To evaluate the efficacy of enhanced external counterpulsation (EECP) in the prevention of contrast-induced nephropathy (CIN) in patients with combined chronic kidney disease (CKD) and diabetes mellitus (DM) by comparing the changes in renal function-related indicators in patients before and after coronary angiography (CAG) or percutaneous coronary intervention (PCI).

**Methods:**

There were 230 subjects consecutively included in the study. Of these, 30 cases with DM underwent rehydration therapy, and 200 cases underwent EECP therapy in addition to rehydration therapy, comprising 53 patients with DM and 147 patients without. All the patients were tested to measure the renal function indicators before and after CAG/PCI.

**Results:**

The postoperative results of blood urea nitrogen (BUN), serum creatinine (Scr), estimated glomerular filtration rate (eGFR), B2 microglobulin, and high-sensitivity C-reactive protein in the three groups showed a statistically significant difference (*P* < 0.05). After EECP therapy, patients with DM showed a significant decrease in BUN (9.1 ± 4.2 vs. 7.2 ± 3.0, *t* = 3.899, *P* < 0.001) and a significant increase in eGFR (41.5 ± 12.7 vs. 44.0 ± 15.6, *t* = −2.031, *P* = 0.047), while the patients without DM showed a more significant difference (*P* < 0.001). Patients with DM showed a lower percentage of elevated Scr (66.7% vs. 43.4%, *P* = 0.042), a higher percentage of elevated eGFR (30.0% vs. 52.8%, *P* = 0.044), and a lower incidence of CIN (16.7% vs. 3.8%, *P* = 0.042) after EECP therapy.

**Conclusion:**

Treatment with EECP can reduce Scr in patients with combined CKD and DM post CAG/PCI, increase eGFR, and decrease the incidence of CIN.

## Introduction

Contrast-induced nephropathy (CIN) is one of the three major causes of hospital-acquired acute kidney injury (AKI) following renal hypoperfusion and drug-induced injuries. It can increase the medical costs of patients, extend the length of their hospital stay, and lead to a poor long-term prognosis. The pathogenesis of CIN is associated with ischemia and anoxia of renal medulla, direct toxicity of contrast medium, oxidative stress, apoptosis, and immunity/inflammation (1), and there are limited therapies for this disease. It is crucial to identify the high-risk population for CIN and take preventive measures. The risk factors of CIN include renal insufficiency, diabetes mellitus (DM), old age, type and dose of contrast medium, and cardio-cerebrovascular comorbidities. Patients with multiple risk factors showed a significant increase in the incidence risk (2, 3).

Enhanced external counterpulsation (EECP) is a non-invasive circulation-assisted device that is very effective in the prevention and treatment of cardiovascular diseases, cardiac rehabilitation, etc. (4) It improves the ischemia of organs and increases the quality of life of patients. Past studies have demonstrated that EECP could decrease renal vascular resistance and renin activity, increase renal blood flow by 20%–30%, and increase estimated glomerular filtration rate (eGFR) by 24% (5). Recent studies suggested possible benefits of EECP in reducing the incidence risk of CIN (6). The study evaluates the efficacy of EECP in the prevention of CIN after coronary angiography (CAG) or percutaneous coronary intervention (PCI) in the high-risk population of patients with chronic kidney disease (CKD) and DM.

## Materials and methods

### Demographics and methods

A total of 230 patients aged 67.2 ± 10.0 years with chronic renal insufficiency undergoing CAG/PCI admitted to Yulin First People’s Hospital from December 2020 to February 2022 were consecutively included as the subjects in the present study. Of these, 36.1% had DM and 62.6% underwent PCI. Thirty cases with DM underwent rehydration therapy 6–12 h before and 6–12 h after CAG/PCI (0.9% normal saline, 1.0–1.5 ml/kg/h; 0.5 ml/kg/h for patients with left ventricular ejection fraction <35%). In addition to rehydration therapy, 200 cases underwent EECP therapy, once per day, 1 h each time, 24 h before and 48–72 h after CAG/PCI; this included 53 patients with DM and 147 patients without. All patients were tested for the renal function indicators before and 48–72 h after CAG/PCI.

### Criteria for diagnosis, inclusion, and exclusion

The diagnosis criteria for CIN was an absolute elevation in serum creatinine (Scr) ≥0.5 mg/dL (44.2 μmol/L) or a 25% elevation relative to baseline value (7) within 48–72 h after iodinated contrast medium was used.

To facilitate earlier identification of acute renal impairment in patients, the concept of AKI in *Kidney Disease: Improving Global Outcomes* was also introduced, which is defined as an absolute elevation in Scr of 0.3 mg/dL (26.5 μmol/L) (8).

The inclusion criteria were as follows: (1) age: ≥18 years old, (2) eGFR <60 ml/min/1.73 m^2^, (3) testing of the renal function indicators within 72 h before the operation, and (4) patient was informed and consent was obtained.

The exclusion criteria were as follows: (1) patients who had used iodinated contrast medium 30 d before inclusion, (2) patients with AKI due to other clear causes, (3) patients requesting withdrawal, (4) patients who failed to receive the re-examination of renal function indicators on time after surgery, (5) patients who underwent hemodialysis within 48 h after surgery, and (6) patients with uremia who received long-term hemodialysis.

### Statistical analysis

The SPSS™ Statistics 22.0 software was used for data analysis. The enumeration data were expressed as [n (%)] and the χ^2^ test was used for comparison between groups. The measurement data fitting the normal distribution were expressed as ± s, one-way analysis of variance was used for comparison between groups, and the paired *t*-test was used for comparison within groups. The measurement data not fitting the normal distribution were expressed as median (first quartile, third quartile) [*M* (*Q_L_, Q_U_*)], and the rank–sum test was used for comparison between groups. A value of *P* < 0.05 denoted a difference was statistically significant.

## Results

### Baseline data

There were no statistically significant differences in gender, age, body mass index (BMI), comorbidities, preoperative renal function indicators, and type of contrast media among the three groups, and the data were comparable (**Table 1**).

**Table 1:** Baseline data of patients.

Baseline data	DM+Rehydration (n = 30)	DM+Rehydration+EECP (n = 53)	Non-DM+Rehydration+EECP (n= 147)	*χ^2^/F/Z*	*P*
Male [n (%)]	21 (70.0%)	37 (69.8%)	117 (79.6%)	2.751	0.253
Age (years)	64.7 ± 10.6	66.5 ± 10.9	68.0 ± 9.5	1.503	0.225
BMI (kg/m^2^)	24.0 ± 2.5	24.5 ± 3.6	23.6 ± 3.5	1.172	0.312
Smoking [n (%)]	11 (36.7%)	10 (18.9%)	46 (31.3%)	3.862	0.145
Hypertension [n (%)]	24 (80.0%)	41 (77.4%)	112 (76.2%)	0.210	0.900
BUN (mmol/L)^a^	10.0 ± 3.9	9.1 ± 4.2	8.2 ± 3.1	5.913	0.052
Scr (μmol/L)	159.3 ± 57.2	160.9 ± 57.8	150.8 ± 40.1	1.103	0.334
eGFR (ml/min/1.73m^2^)	41.8 ± 12.0	41.5 ± 12.7	43.7 ± 11.1	0.885	0.414
PCI [n (%)]	21 (70.0%)	31 (64.2%)	92 (62.6%)	0.596	0.742
Type of contrast medium					
Isotonic [n (%)]	12 (40.0%)	24 (45.3%)	55 (37.4%)	1.011	0.603
Hypotonic [n (%)]	18 (60.0%)	29 (54.7%)	92 (62.6%)		
Total rehydration amount (ml)	1000.0 (687.5,1000.0)	1000.0 (1000.0,1000.0)	1000.0 (1000.0,1000.0)	5.565	0.062
Duration of surgery (min)	55.0 (25,73.5)	50.0 (24,80.5)	50.0 (25.0,67.0)	1.461	0.482

BMI, body mass index; BUN, blood urea nitrogen; Scr, serum creatinine; eGFR, estimated glomerular filtration rate; PCI, Percutaneous coronary intervention; ^a^ denotes heterogeneity of variance, rank-sum test; ^b^ denotes M(Q_L_,Q_U_).

### Comparison of the postoperative renal function indicators among the three groups

Postoperative blood urea nitrogen (BUN), Scr, eGFR, B2 microglobulin, and high-sensitivity C-reactive protein in the three groups showed statistically significant differences (*P* < 0.05). As shown in the pairwise comparison of the three groups, no differences were observed between DM + Rehydration and DM + Rehydration + EECP or between DM + Rehydration + EECP and non-DM + Rehydration + EECP (**Table 2**).

**Table 2:** Comparison of postoperative renal function indicators among three groups.

Item	DM+Rehydration (n = 30)	DM+Rehydration+EECP (n = 53)	Non-DM+Rehydration+EECP (n = 147)	*F/Z*	*P*
BUN(mmol/L)^a^	9.5 ± 6.8	7.2 ± 3.0	6.5 ± 2.4	11.884	0.003
Scr(μmol/L)	164.5 ± 63.3	157.6 ± 69.5	142.4 ± 39.2	3.363	0.036
eGFR(ml/min/1.73m^2^)	40.7 ± 13.2	44.0 ± 15.6	47.3 ± 13.2	3.302	0.039
β2-microglobulin(mg/L)	4.3 ± 1.6	4.1 ± 1.8	3.2 ± 1.2	24.835	<0.001
Cystatin(mg/L)	2.0 ± 0.6	1.8 ± 0.6	1.5 ± 0.4	11.793	<0.001
Retinol-binding protein(mg/L)	48.1 ± 13.5	49.4 ± 15.8	46.9 ± 13.0	0.564	0.570
Hs-CRP(mg/L)^a^	35.5 ± 48.4	19.5 ± 31.7	14.9 ± 23.2	10.705	0.005

Hs-CRP, Hypersensitive C-reactive protein.

a denotes heterogeneity of variance, rank-sum test.

### Comparison of the renal function levels in the three groups before and after the operation

There was no significant difference in BUN, Scr, and eGFR in the DM + Rehydration group before and after the operation (*P* > 0.05). In the DM + Rehydration + EECP treatment group, BUN decreased significantly (9.1 ± 4.2 vs. 7.2 ± 3.0, *t* = 3.899, *P* < 0.001) and eGFR increased significantly (41.5 ± 12.7 vs. 44.0 ± 15.6, *t* = −2.031, *P* = 0.047) after the operation, and these differences were more significant between patients in the non-DM group (*P* < 0.001) (**Table 3**).

**Table 3:** Comparison of renal function before and after surgery.

Group	Item	Preoperative	Postoperative	*t*	*P*
DM+Rehydration (n = 30)	BUN (mmol/L)	10.0 ± 4.0	9.5 ± 6.8	0.374	0.711
	Scr (μmol/L)	159.3 ± 57.2	164.5 ± 63.3	-0.809	0.425
	eGFR (ml/min/1.73m^2^)	41.8 ± 12.0	40.7 ± 13.2	0.564	0.577
DM+Rehydration+EECP (n = 53)	BUN (mmol/L)	9.1 ± 4.2	7.2 ± 3.0	3.899	<0.001
	Scr (μmol/L)	160.1 ± 57.8	157.6 ± 69.5	0.769	0.445
	eGFR (ml/min/1.73m^2^)	41.5 ± 12.7	44.0 ± 15.6	-2.031	0.047
Non-DM+Rehydration+EECP (n = 147)	BUN (mmol/L)	8.2 ± 3.1	6.5 ± 2.4	8.315	<0.001
	Scr (μmol/L)	150.8 ± 40.1	142.4 ± 39.2	5.108	<0.001
	eGFR (ml/min/1.73m^2^)	43.7 ± 11.1	47.3 ± 13.2	-4.922	<0.001

### Comparison of the CIN risks in the three groups

The changes of postoperative Scr and eGFR and the incidences of AKI and CIN showed a statistically significant difference in the three groups (*P* < 0.05). The pairwise comparison demonstrated patients with DM had a lower rate of increased Scr (66.7% vs. 43.4%, *P* = 0.042), a higher rate of increased eGFR (30.0% vs. 52.8%, *P* = 0.044), and a lower incidence of CIN (16.7% vs. 3.8%, *P* = 0.042) after EECP therapy. Among patients undergoing EECP therapy, the rate of AKI was higher in patients with DM (13.2% vs. 2.0%, *P* = 0.001) (**Table 4**).

**Table 4:** Comparison of incidence of contrast-induced nephropathy.

Item	DM+Rehydration (n = 30)	DM+Rehydration+EECP (n = 53)	Non-DM+Rehydration+EECP (n = 147)	*χ^2^*	*P*
Change of BUN					
Increase	10 (33.3%)	15 (28.3%)	32 (21.8%)	2.245	0.325
Decrease	20 (66.7%)	38 (71.7%)	115 (78.2%)		
Change of Scr					
Increase	20 (66.7%)	23 (43.4%)	51 (34.7%)	10.721	0.005
Decrease	10 (33.3%)	30 (56.6%)	96 (65.3%)		
Change of eGFR					
Increase	9 (30.0%)	28 (52.8%)	94 (63.9%)	12.189	0.002
Decrease	21 (70.0%)	25 (47.2%)	53 (36.1%)		
AKI [n (%)]	7 (23.3%)	7 (13.2%)	3 (2.0%)	19.906	<0.001
CIN [n (%)]	5 (16.7%)	2 (3.8%)	1 (0.7%)	18.984	<0.001

AKI, Acute kidney injury; CIN, Contrast induced nephropathy.

## Discussion

The definition of CIN is an absolute elevation in Scr ≥0.5 mg/dL (44.2 μmol/L) or a 25% elevation relative to baseline within 48–72 h after the use of iodinated contrast medium. The incidence of CIN reported in different studies is associated with the definition, potential renal impairment, type and dose of contrast medium, and other concomitant factors (9). The incidence of CIN is <3% in patients with normal renal function but up to 50% in patients with CKD. Following the use of isotonic and hypotonic contrast media, the incidence of CIN may be overestimated, but in patients adopting transarterial administration and with eGFR <30 mL/min/1.73 m^2^, the incidence of CIN still increased significantly (10).

A high-risk factor for CIN is DM, with the mechanism possibly associated with increased oxidative stress, forceful contraction of renal blood vessels, and signaling route induced apoptosis, under a high glucose condition (11). The incidence of CIN was only 2% in patients without DM and with normal renal function, 16% in patients with DM and normal renal function, and 38% in patients with DM and renal insufficiency (12). Up to 56% of CIN cases developed to irreversible renal failure.

Rehydration therapy is the standard care for CIN, but in the present study, the incidence of CIN was 16.7% in patients with DM after standard rehydration therapy, higher than the 6.77% from previous studies (13) and similar to the result of Liang (11.67%) (14) due to the subjects of the study having old age and a high PCI rate. However, the effect of normal saline on the prevention of CIN was contested in a study that concluded that an intravenous drip of normal saline could not reduce the risk of CIN (15). That study disrupted the existing knowledge and suggests further consideration is needed.

The data on the effect of sodium bicarbonate on preventing CIN was contradictory. The efficacy of statins in the prevention of CIN has been widely acknowledged. As suggested in a meta-analysis, rosuvastatin could decrease the incidence rate of CIN by 47% (odds ratio [OR]: 0.53, 95% confidence interval [CI]: 0.40–0.71, *P* < 0.0001) (16). Sulfhydryl in n-acetylcysteine is a good antioxidant and agent for removing oxygen radicals at a low cost and with no adverse reactions, so it is one option for the prevention of CIN (17). Currently, no conclusive evidence is available to prove preventive hemodialysis can prevent contrast-induced renal impairment, and the risk of invasive operation cannot be neglected, so most scholars do not recommend preventive hemodialysis (7).

Treatment with EECP is a safe, non-invasive extracorporeal circulation method that is widely used for cardiac-cerebral vascular diseases to reduce angina symptoms and the use of nitrates, increase exercise tolerance, and improve myocardial ischemia (18). It could also improve the quality of life of patients with cardiac failure (19), and have a positive effect on the hemodynamics of the coronary and carotid arteries (20, 21). However, there are few studies on the effect of EECP on renal functions. Werner reported that EECP could effectively increase renal excretion, renal blood flow, and eGFR (5). Rua discovered that serum cystatin significantly decreased from 1.00 to 0.94 mg/L (*P* < 0.001) and eGFR increased from 70.47 to 76.27 mL/min/1.73 m^2^ (*P* = 0.006) after EECP therapy (22).

A recent study suggested that EECP could increase the clearance rate of the contrast medium and reduce the incidence of CIN (6). First, EECP increases renal blood flow and decreases the plasma levels of renin (5). The increase in renal blood flow is due to not only an increase in renal perfusion pressure caused by increased blood return to the heart, but also an increase in nitric oxide. A previous study has shown that EECP increases the plasma concentrations of nitric oxide and decreased plasma levels of endothelin-1, and improves the renal function (23). Secondly, the increase in blood flow shear stress induced by EECP improves the function and morphology of the vascular endothelium and reduces oxidative stress and inflammatory responses. Meanwhile, increased renal blood flow shear stress induces the expression of nuclear factor erythroid-2-related factor 2 (Nrf2) in the proximal tubular epithelium (24), activates the Nrf2/Sirtuin-3 (Sirt3)/superoxide dismutase 2 (SOD2) signaling pathway, and improves the contrast-induced renal injury (25). Lastly, the increase of atrial natriuretic peptide induced by EECP has a diuretic effect (26), which improves the fluid shear stress of the kidney and reduces the viscosity of the contrast medium, thereby alleviating kidney damage caused by the contrast medium.

As demonstrated in the present study, after EECP treatment, the incidence of CIN in patients with DM decreased to 3.8%, dropping by 77.2% as compared with those treated solely with rehydration, and eGFR also increased by 2.5 ml/min/1.73m^2^. Among patients without DM, EECP showed a more significant effect, reducing Scr by 8.4 μmol/L and increasing eGFR by 3.6 ml/min/1.73 m^2^ after the operation. It may become a new choice for the prevention of CIN.

However, there are some limitations in the present study. The exact dose of the contrast medium was not recorded and was instead evaluated indirectly by the duration of the operation. In this study, not all the total amount of contrast media was recorded due to different habits of interventional surgeons in writing surgical records. Since the eGFR of the recruited patients was less than 60 mL/min/1.73m^2^, the risk of CIN was high. To ensure the safety of patients, only non-ionic contrast media were used, including iodixanol, omnipac, and ioversol. Lastly, the sample size was small, and the mechanism for CIN prevention by EECP was not further examined.

## Conclusion

It was demonstrated that EECP can reduce the Scr level in patients with combined CKD and DM post CAG/PCI, increase eGFR, and decrease the incidence of CIN.

## Data availability statement

The original contributions presented in the study are included in the article/supplementary material. Further inquiries can be directed to the corresponding author.

## Ethics statement

The studies involving human participants were reviewed and approved by the Ethics Committee of Yulin First People’s Hospital. The patients/participants provided their written informed consent to participate in this study.

## Author contributions

Conception and design of the research: C-MZ. Acquisition of data: C-MZ, X-JZ, Z-JW, JB, and S-YQ. Analysis and interpretation of the data: C-MZ and Y-MZ. Statistical analysis: C-MZ and Y-MZ. Obtaining financing: C-MZ and Y-YL. Writing of the manuscript: C-MZ. Critical revision of the manuscript for intellectual content: Y-YL. All authors read and approved the final draft.

## Funding

This work was supported by Health Committee of Guangxi (No.Z20210519).

## Acknowledgments

We would like to acknowledge the hard and dedicated work of all the staff that implemented the intervention and evaluation components of the study.

## Conflict of interest

The authors declare that the research was conducted in the absence of any commercial or financial relationships that could be construed as a potential conflict of interest.

## Publisher’s note

All claims expressed in this article are solely those of the authors and do not necessarily represent those of their affiliated organizations, or those of the publisher, the editors and the reviewers. Any product that may be evaluated in this article, or claim that may be made by its manufacturer, is not guaranteed or endorsed by the publisher.
